# Supplementary material for: The causal relationship between the human gut microbiota and pyogenic arthritis: a Mendelian randomization study
Source: Front Cell Infect Microbiol. 2024 Nov 26;14:1452480. doi: 10.3389/fcimb.2024.1452480 (PMC11629706; doi:10.3389/fcimb.2024.1452480)
Supplement: Supplementary file 1 [file DataSheet1.zip › mendelian test/ebi-a-GCST90027526.csv_forest.pdf]

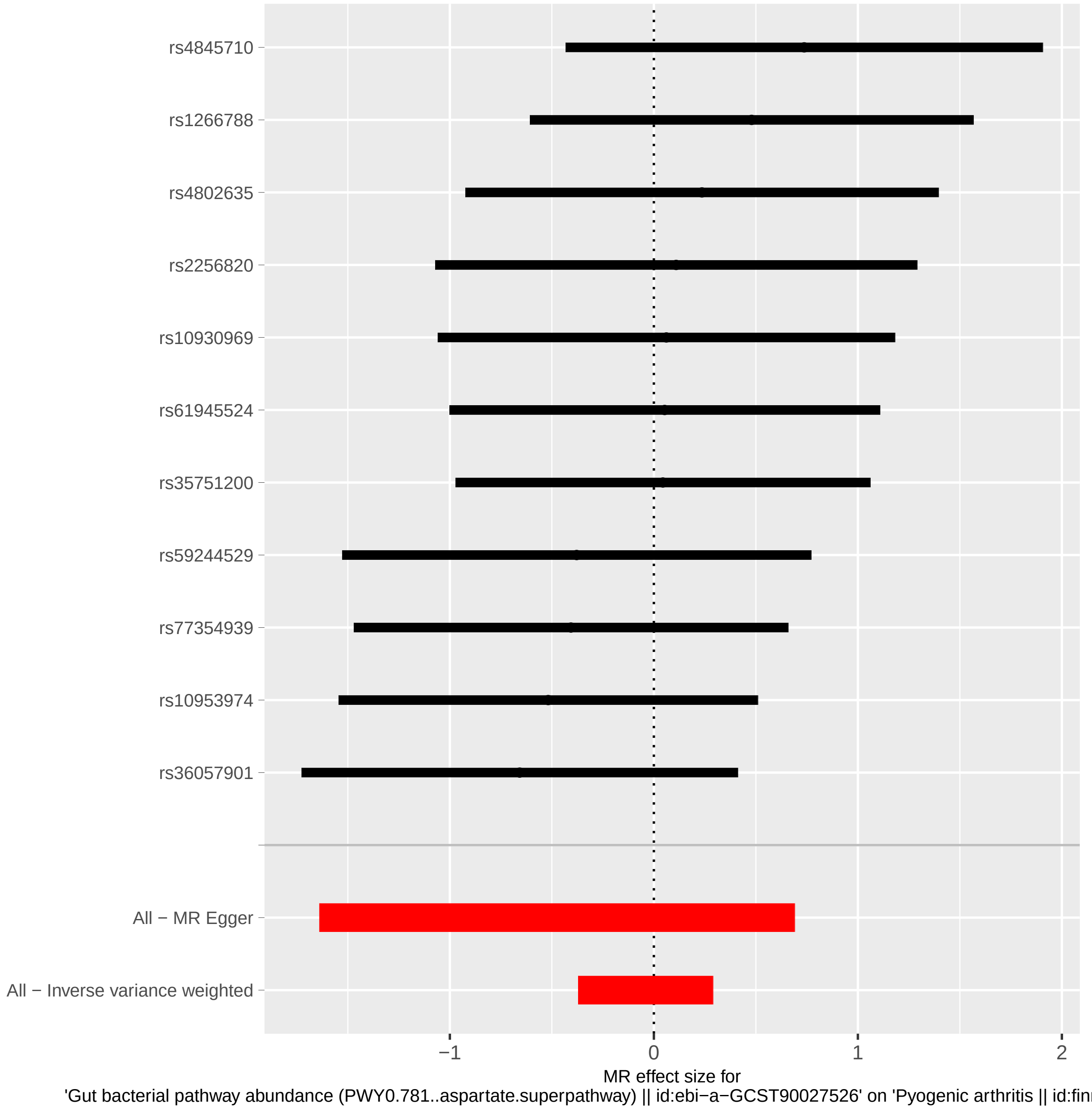

rs4845710
rs1266788
rs4802635
rs2256820
rs10930969
rs61945524
rs35751200
rs59244529
rs77354939
rs10953974
rs36057901
All − MR Egger
All − Inverse variance weighted
−1
0
1
2
MR effect size for
'Gut bacterial pathway abundance (PWY0.781..aspartate.superpathway) || id:ebi−a−GCST90027526' on 'Pyogenic arthritis || id:fin
